# Involvement of Adenosine A_2_A Receptors in Anxiety‐Like Behaviors in Tetrahydrocannabinol‐Treated Mice

**DOI:** 10.1002/brb3.71126

**Published:** 2026-01-28

**Authors:** Burçin Ün, Zeki Akarsakarya, Özlem Yorulmaz Özü, Nermin Seda Ilgaz, Mehmet Bertan Yılmaz, Deniz Seçilmiş, Mehmet Ata Seçilmiş

**Affiliations:** ^1^ Department of Addiction, Institute of Addiction and Forensic Sciences Çukurova University Adana Turkey; ^2^ Dentistry Faculty Basic Sciences Istanbul Kent University Istanbul Turkey; ^3^ Department of Pharmacology, Medical Faculty Çukurova University Adana Turkey; ^4^ Department of Medical Biology, Medical Faculty Çukurova University Adana Turkey; ^5^ Department of Cell and Molecular Biology Karolinska Institutet Stockholm Sweden

**Keywords:** adenosine A_2_A receptors, anxiety‐like behaviors, hippocampus, tetrahydrocannabinol

## Abstract

**Background:**

Previous studies have suggested that adenosinergic system in the central nervous system may play a role in both behavioral changes and the physiopathology induced by Δ^9^‐tetrahydrocannabinol (THC), and this is thought to be mediated by adenosine A_2_A receptors (A_2_ARs). However, the contribution of the adenosinergic system to the anxiety‐like behaviors in response to THC in mice is not well understood.

**Aims:**

In this study, we aimed to investigate the possible role of the adenosinergic system in THC‐treated mice.

**Methods:**

For that purpose, we combined behavioral tests and molecular analyses to investigate the effects of THC in relation with the agonist and antagonist of the adenosinergic system, CGS‐21680 (CGS) and istradefylline, respectively, on both anxiety‐like behaviors and hippocampal gene expression.

**Results:**

The results demonstrated that THC induced anxiety‐like behavior, and gene expression patterns indicated a significant interaction between the adenosinergic and cannabinoidergic systems. Notably, the data suggest that THC plays a predominant role in this molecular interplay, with its effects being partially modulated by changes in the expression of both cannabinoidergic and adenosinergic receptors, CB_1_R and A_2_AR, respectively.

**Conclusion:**

These findings contribute to the understanding of THC's complex pharmacological actions, highlighting the importance of receptor cross talk in modulating anxiety and other behavioral outcomes.

## Graphical abstract

This study evaluated anxiety‐like behavior in mice across treatment and control groups using open field and elevated plus maze tests, followed by hippocampal receptor gene expression analysis. The combined behavioral and molecular data identify potential receptor‐level mechanisms underlying the observed treatment‐associated behavioral changes.

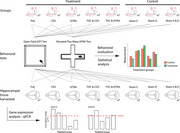

## 1 Introduction

Cannabis is one of the most widely used substances globally and is known for its addictive properties (Shao et al. 2023). Its use is associated with a range of serious issues, including cognitive impairment in areas such as learning and memory, mood disorders such as psychosis and paranoia, as well as social difficulties, job loss, and an increased likelihood of engaging in violent or aggressive behavior (Pertwee 2005). However, compounds derived from cannabis, such as Δ^9^‐tetrahydrocannabinol (THC) and cannabidiol (CBD), have therapeutic benefits, including pain relief, anxiety reduction, and improved sleep (Stollenwerk et al. 2021).

The relationship between THC and anxiety is complex and depends significantly on the dose, individual characteristics, and frequency of use (Sharpe et al. 2020). Although THC might act as an anxiolytic (anxiety‐reducing) agent in low doses, possibly by modulating the brain's endocannabinoid system, which plays a crucial role in regulating emotional responses and stress (Haller et al. 2004), it has also been shown that high doses of THC are often associated with increased anxiety and even paranoia, primarily due to overstimulation of cannabinoid receptors in brain (D'Souza et al. 2004). Therefore, it is crucial to develop a clear and comprehensive understanding of the mechanisms underlying the behavioral changes associated with cannabis use.

Upon ingestion, cannabis’ psychoactive constituent, THC, rapidly enters the brain and other organs, binding to specific receptors, thus demonstrating its effects in different regions of the brain, such as the hippocampus, amygdala, and striatum (FFFLM and Karch 2007). Although numerous studies have focused on the main localization and function of cannabinoidergic receptor CB_1_R, which play a role in THC's effects, its interaction with other systems such as the adenosinergic complicates the understanding of the mechanisms involved in THC's broad impact across the brain (Koob 1992; Margolis et al. 2012; Pariyadath et al. 2016; Ranade 2021; Stahl 2013; Stollenwerk et al. 2021; Volkow et al. 2019; Wise and Robble 2020).

Adenosine, a molecule synthesized endogenously within and outside cells, is formed by the addition of a pentose ring to adenine, which is one of the purine bases contributing to the formation of genetic material (Cobbin et al. 1974). Its most crucial source is adenosine triphosphate (ATP), regularly utilized within the cell and stored in vesicles in presynaptic nerve terminals alongside neurotransmitters such as dopamine, serotonin, norepinephrine, and acetylcholine. Upon cell stimulation, ATP is released into the synaptic cleft along with neurotransmitters, acting as a neuromodulator (Fredholm et al. 1982; Sachdeva and Gupta 2013). The adenosinergic system receptors acting as neuromodulators in the central nervous system (CNS) are suggested to play a role in both the behavioral changes induced by THC and the pathophysiology of anxiety, with the mediation of adenosine A_2_A receptor (A_2_AR) (R. M. Brown and Short 2008; Chen et al. 2003; Filip et al. 2012; Knapp et al. 2001; Munzar et al. 2002). Furthermore, it has been reported that the cataleptic response to THC is diminished in the pharmacological inhibition of A_2_AR (Yamada et al. 2014) or in the absence of these receptors (Stollenwerk et al. 2021). It has previously been shown that cannabinoid CB_1_R and adenosine A_2_AR are expressed in the hippocampus (Aso et al. 2019); however, the role of these receptors in the behavioral effects of THC and the involvement of the adenosinergic system are still not completely understood.

Therefore, in this study, we investigated the potential role of A_2_AR in anxiety‐like behavior observed in mice exposed to a high dose of THC. Behavioral experiments were conducted on mice to assess the effects of THC, and the expression levels of A_2_AR and CB_1_R genes were examined by isolating the hippocampal tissue from each mouse.

## 2 Materials and Methods

### 2.1 Animals

A total of 56 male Swiss albino mice (8–10 weeks old) were used in this study. During the experiment, the animals were housed under controlled conditions: A 12‐h light/dark cycle maintained with an automatic control system, and room temperature (22°C ± 1°C) and humidity (40%–60%) regulated by air conditioning. Tap water and food pellets were provided ad libitum.

### 2.2 Experimental Groups

Mice were randomly assigned to one of the eight experimental groups (*n* = 7 per group): THC, CGS, ISTRA, THC & CGS, THC & ISTRA, SHAM‐E, SHAM‐D, and SHAM‐E & ‐D. SHAM‐E is the control group for THC, and SHAM‐D is the control group for the CGS and ISTRA groups. SHAM‐E& ‐D is the control group for the combination treatment groups THC & CGS and THC & ISTRA.

### 2.3 Drugs

The following drugs were used in this study: (−)‐Δ9‐tetrahydrocannabinol (THC) (ETNA C9743.21‐100K‐ME), ethanol (Sigma‐Aldrich), adenosine A_2_AR agonist CGS‐21680 (Sigma‐Aldrich), adenosine A_2_AR antagonist Istradefylline‐KW6002 (CAYMAN), and dimethyl sulfoxide (DMSO) (Sigma‐Aldrich).

#### 2.3.1 Drug Administration

All mice were treated over a period of 5 days, receiving daily injections at 9 a.m. either intraperitoneally (i.p.) or subcutaneously (s.c.), depending on the group. The route of administration (i.p. or s.c.) was chosen according to drug formulation and solvent tolerability. In the THC group, THC was administered at a dose of 10 mg/kg/day (i.p.) and dissolved in 7.2% ethanol and saline; the i.p. route was selected because it was the most commonly preferred THC administration route in previous studies (Sharpe et al. 2020). In the CGS and ISTRA groups, CGS‐21680 was administered at a dose of 2.5 mg/kg/day (s.c.), whereas Istradefylline‐KW6002 was administered at a dose of 3 mg/kg/day (s.c.), both dissolved in 5% DMSO and saline. The s.c. route was chosen in these groups because of the use of DMSO as the solvent: DMSO has been reported as invasive and to produce local and systemic adverse effects when administered i.p. at higher volumes or concentrations; therefore, s.c. administration helps to minimize peritoneal irritation and administration‐related confounds (Jacob and de la Torre 2009; Santos et al. 2003). In combination groups (THC & CGS and THC & ISTRA), mice first received THC (10 mg/kg, i.p.), followed 30 min later by CGS‐21680 (2.5 mg/kg, s.c.) and Istradefylline‐KW6002 (3 mg/kg, s.c.), respectively. The use of different routes is expected to reduce cumulative peritoneal stress by preventing the administration itself from meaningfully contributing to anxiety‐like behavior in the combined treatment groups. The control treatments reflected the solvent used for the experimental drugs. All drugs were dissolved according to the manufacturers’ specified methods.

### 2.4 Behavioral Experiments

Behavioral tests were conducted to assess exploratory and anxiety‐like behaviors, starting on the third day of drug administration. The first test, the open field (OF), was performed 30 min after the last daily injection on Day 3, and the second test, the elevated plus maze (EPM), was conducted on Day 5 after the final injection. To acclimate the mice to the testing environment, they underwent a 3‐day hand adaptation before the experiments began. During the tests, exploratory and anxiety‐like behaviors were evaluated.

#### 2.4.1 Open Field Test (OFT)

The OFT was conducted using a black opaque Plexiglass apparatus measuring 60 cm × 60 cm × 24 cm with an open top and surrounded by 1 cm thick walls on all sides. The area next to the walls was defined as the periphery, whereas the central region was classified as the center. Mice were brought to the experiment room 3 min before the test to acclimate to the environment, which was maintained at room temperature of 22°C ± 1°C with light intensity set to 100 lx. Mice were placed into the field from any corner, and their behavior was recorded for 5 min using a video camera. After each test, the arena was cleaned with 70% alcohol. The frequency of entries into the center area and rearing events were measured to evaluate exploratory and anxiety‐like behaviors.

#### 2.4.2 Elevated Plus Maze (EPM) Test

The EPM apparatus consisted of two perpendicular open arms (30 cm × 5 cm) and two perpendicular closed arms (30 cm × 5 cm × 15 cm), connected by a central platform (center, 5 cm × 5 cm). The maze apparatus was made of black acrylic and elevated 40 cm above the ground. After the 3‐min adaptation period, each animal was placed in the center of the maze facing an open arm, and their movement was recorded for 5 min. The time spent in open/closed arms and in the center is used to evaluate anxiety‐like behaviors. All parameters were manually counted after video recordings. The arena was cleaned with 70% alcohol solution after each test.

### 2.5 Statistical Analysis

For each behavioral measure, including the frequency of center entries and rearing events from the OFT, and the time spent in the open/closed arms and center from the EPM test, we first detected outliers within each group using the interquartile range (IQR) method and then performed an unpaired two‐sided Mann–Whitney *U* test between each treatment group and its corresponding control group, excluding the outliers detected within each sample from the statistical comparison. Statistical significance thresholds were set at *p* < 0.05 (*), *p* < 0.01 (**), and *p* < 0.001 (***). The selection of a nonparametric test was made due to the small sample size (*n* = 7 per group). All statistical analyses were performed in R (v4.3.3), employing *dplyr()* for data handling, *base* packages for computation, and *ggplot2()* for visualization.

### 2.6 Gene Expression Analysis

Following the EPM test, the mice were sacrificed through the cervical dislocation method, and their brains were carefully extracted and placed in Petri dishes containing ice‐cold 0.1 M sodium phosphate buffer (PBS) at +4°C. The hippocampal tissues were then isolated as described by Spijker (2011). RNA isolation from the hippocampal tissues was performed using the Hibrigen MG‐HBRZ‐01‐100 kit, according to the manufacturer's protocol. The isolated RNAs were then reverse‐transcribed to complementary DNAs (cDNA) for quantitative real‐time PCR (qPCR) using abm's OneScript Plus cDNA Synthesis Kit. Gene expression analysis was performed using EnTurbo SYBR Green PCR SuperMix kit and a Bio‐Rad DX Real‐Time Systems device to quantify the mRNA expression of the target receptor genes, *Adora2a* for A_2_AR and *Cnr1* for CB_1_R, with *Actb* (beta‐actin) serving as the housekeeping gene. The primers used for real‐time PCR are provided in Table 1. Upon completion of the qPCR analysis, the cycle threshold (Ct) values for each sample were recorded. The difference between the Ct value of the target gene and the Ct value of the housekeeping gene is calculated as $\Delta Ct_{G,i,T}$ for each target gene *G* and animal *i (I = *1*, …, n, n = *7) in treatment group *T* (Equation 1), yielding normalized gene expression levels to the housekeeping gene within each sample. Statistical analyses were performed on these ΔCt values. For each target gene, comparisons between each treatment group and its corresponding control were evaluated using a two‐sided, unpaired Mann–Whitney *U* test after exclusion of identified outliers via the IQR method. Statistical significance thresholds were set at *p* < 0.05 (*), *p* < 0.01 (**), and *p* < 0.001 (***). The choice of a nonparametric test was due to the small sample size. As the treatment groups are independent, the ΔCt values were then averaged across all animals in each treatment group *T* and its corresponding control group *C(T)*, for each gene *G* $\bar{\Delta Ct_{G,T}}$ for treatment *T* (Equation 2), and $\bar{\Delta Ct_{G,C(T)}}$ for control treatment *C(T)* (Equation 3). Finally, the relative difference in normalized gene expression between each treatment group *T* and its corresponding control group *C(T)* was computed for each target gene *G* (Equations 4 and 5) (Livak and Schmittgen 2001):

(1) $$\Delta Ct_{G,i,T}=Ct_{G,i,T}-Ct_{ACTB,i,T}$$

(2) $$\bar{\Delta Ct_{G,T}}=\frac{1}{n}\sum_{i=1}^{n}\Delta Ct_{G,i,T}$$

(3) $$\bar{\Delta Ct_{G,C\left(T\right)}}=\frac{1}{n}\sum_{i=1}^{n}\Delta Ct_{G,i,C\left(T\right)}$$

(4) $$\Delta \Delta Ct_{G,T}=\bar{\Delta Ct_{G,T}}-\bar{\Delta Ct_{G,C\left(T\right)}}$$

(5) $$\mathrm{Relative}\;\mathrm{chang}e_{G,T}=2^{-\Delta \Delta Ct_{G,T}}$$

**TABLE 1:** Primers used for real‐time PCR.

Gene	Primer sequence
***Cnr1***	F:GTGCTGTTGCTGTTCATTGTG R:CTTGCCATCTTCTGAGGTGTG
***Adora2a***	F: CCGAATTCCACTCCGGTACA R: CAGTTGTTCCAGCCCAGCAT
***Actb***	F: TGACCGAGCGTGGCTACA R:CATAGCACAGCTTCTCTTTGATGTC

All computational analyses were performed in R (v4.3.3), employing *dplyr()* for data handling, *base* packages for computation, and *ggplot2()* for visualization.

## 3 Results

**FIGURE 1:**
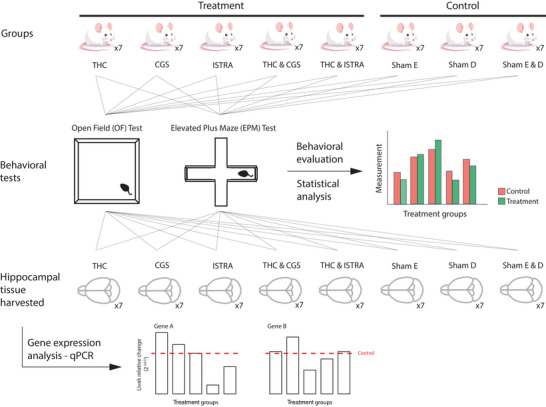
Workflow of the present study. A total of 56 mice were divided into eight study groups: five treatment groups and three control groups. Anxiety‐like behavior in mice in each study group was measured by the open field (OF) and elevated plus maze (EPM) tests, and the statistical significance of the difference between behavioral patterns of each treatment group and their corresponding control group is computed. After the behavioral tests are completed, the hippocampal tissues of these mice's brains are harvested and gene expression analysis of the key receptors of the systems of interest is performed to reveal the possible molecular mechanisms behind the observed behavioral patterns.

In this study, we aimed to investigate the effect of THC in a possible interplay between the adenosinergic and cannabinoidergic systems in the modulation of anxiety‐like behaviors in mice (Figure 1). As THC has previously been shown to exhibit an anxiolytic or anxiogenic effect depending on the administration dose (Sharpe et al. 2020), we administered it at high doses (10 mg/kg) to ensure anxiety‐like behavior in mice. To be able to thoroughly assess the effect of THC, we also included treatment groups for both the agonist (CGS) and antagonist (ISTRA) of the adenosinergic system receptor individually and in combination with THC (see Section 2).

### 3.1 Assessing the Anxiety‐Like Behavior in Mice

#### 3.1.1 THC and/or CGS Induce Anxiety

**FIGURE 2:**
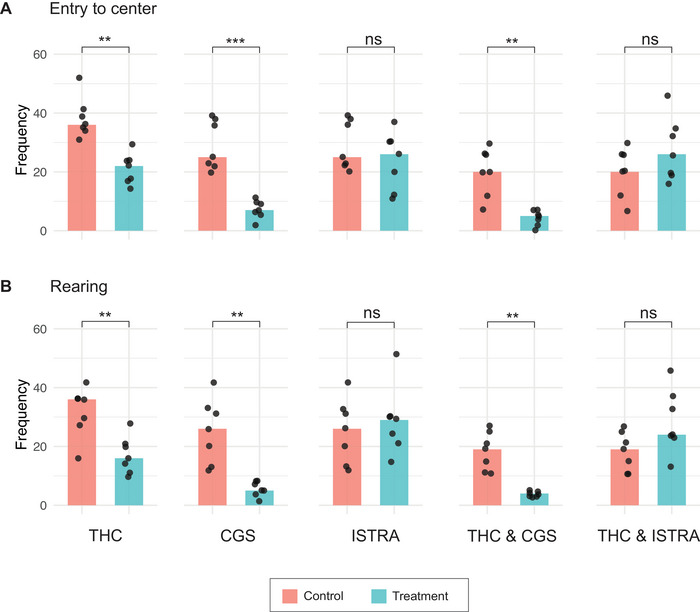
Open field test (OFT) analysis of exploratory behavior in mice across treatment groups. Assessment of exploratory behavior in terms of frequencies of (A) entry to center and (B) rearing events during the OFT. Bar plots represent the median frequency of these behaviors, with individual data points shown as dots. Data are presented for each treatment group compared to their corresponding control group (SHAM‐E for THC; SHAM‐D for CGS and ISTRA; SHAM‐E & D for THC & CGS, and THC & ISTRA). Statistical significance of the test is shown as ns for nonsignificant, *p < 0.05, **p < 0.01, and ***p < 0.001

We first assessed the impact of the treatments on exploratory and anxiety‐like behaviors using the OF and EPM tests. In the OF test, the frequency of entries into the center zone and rearing events were used as indicators of exploratory behavior (Figure 2). Mice treated with THC showed a significant reduction in center entries (1.64** fold decrease) and rearing events (2.25** fold decrease) compared to their control group, SHAM‐E. A similar trend was also observed in the CGS group (3.57*** fold decrease in center entries and 5.2** fold decrease in rearing events), and in the combined treatment group of THC & CGS (4** fold decrease in center entries and 4.75** fold decrease in rearing), indicating anxiety.

**FIGURE 3:**
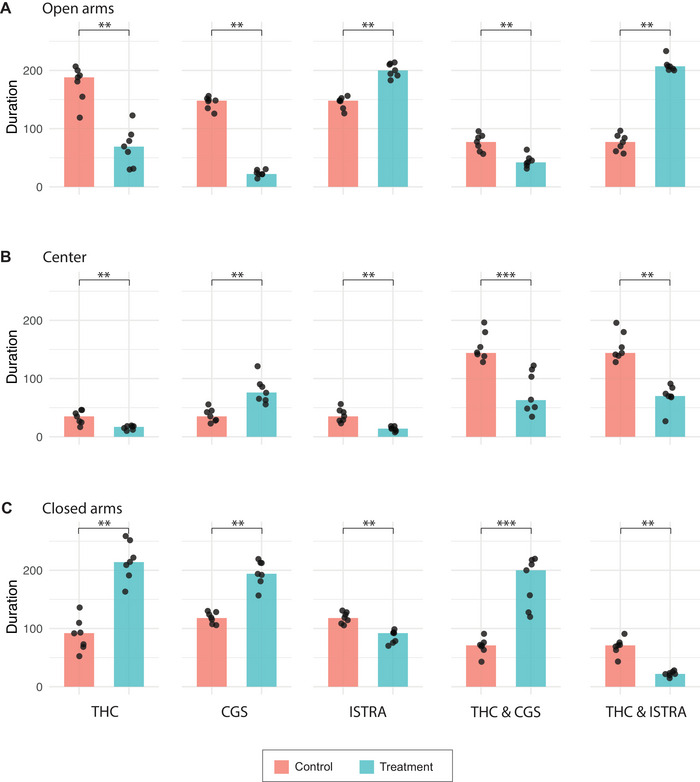
Elevated plus maze test (EPM) analysis of exploratory behavior in mice across treatment groups. Assessment of exploratory behavior in terms of durations of time spent in (A) open arms, (B) center, and (C) closed arms during the EPM. Bar plots represent the median frequency of these behaviors, with individual data points shown as dots. Data are presented for each treatment group compared to their corresponding control group (SHAM‐E for THC; SHAM‐D for CGS and ISTRA; SHAM‐E & D for THC & CGS and THC & ISTRA). Statistical significance of the test is shown as ns for nonsignificant, *p < 0.05, **p < 0.01, and ***p < 0.001.

In the EPM test (Figure 3), behavioral assessment was made by the time spent in the open arms, center, and closed arms of the maze. An increased time spent in the closed arms is attributed to anxiety. Mice treated with THC and/or CGS spent significantly less time in open arms (2.72** fold decrease for THC, 6.73** fold decrease for CGS, and 1.83** fold decrease for THC & CGS) and more time in closed arms (2.33** fold increase for THC, 1.64** fold increase for CGS, and 2.82*** fold increase for THC & CGS) compared to their control groups. Although time spent in the center followed the same trend as in the open arms for the THC (2.06** fold decrease in center time) and THC & CGS (2.29*** fold decrease in center time) groups, the mice in the CGS group spent more time not only in closed arms but also in the center zone (2.17** fold increase in center time) compared to their control groups.

Behavioral results from both the OF and EPM tests strongly suggest that THC and/or CGS induce anxiety‐like behavior, as demonstrated by reduced exploratory activity in the OF test (Figure 2) and a shift in time spent from open arms to closed arms in the EPM test (Figure 3).

#### 3.1.2 Istradefylline Reverses THC's Anxiety‐Inducing Effect

In contrast to the anxiety‐inducing effects of THC and/or CGS, the ISTRA group, where mice were treated with istradefylline, displayed an anxiolytic effect, spending significantly more time in the open arms compared to their control group, SHAM‐D (1.35** fold increase) (Figure 3A). This suggests that although istradefylline did not induce anxiety, it may have provided a calming or relaxing effect. A similar anxiolytic effect was observed also in the THC & ISTRA group (2.69** fold increase in time in open arms and 3.23** fold decrease in time in closed arms) (Figure 3A,C), suggesting istradefylline did not only prevent the anxiogenic effects of THC but may have reversed them. This observation from the EPM was not confirmed by the OF test. Although the results of the OF test suggested a potential increase in exploratory behavior in the ISTRA (1.04 fold increase in center entries and 1.12 fold increase in rearing events) and THC & ISTRA (1.3 fold increase in center entries and 1.26 fold increase in rearing events) groups, this increase was not statistically significant (Figure 2). As the OF test was performed on Day 3 of the experiment and the EPM test was performed on Day 5, the reason for a present but nonsignificant difference in the OF test could be due to insufficient amount of time for istradefylline to start showing its effects on mice's behavior for the OF test.

### 3.2 Gene Expression Analysis of A_2_AR and CB_1_R

The behavioral results demonstrate that THC and/or CGS induce significant anxiety‐like behaviors, whereas ISTRA not only prevents but may also reverse THC's effects. Given the distinct behavioral outcomes observed across different treatment groups confirming THC's anxiogenic effect, we next investigated the molecular mechanisms underlying the interplay between the adenosinergic and cannabinoidergic systems. Specifically, we focused on expression levels of A_2_AR and CB_1_R in the hippocampus, as these genes are key players in neuromodulatory pathways associated with anxiety and cannabinoid signaling, which may help uncover the molecular correlates of the anxiety‐modulating effects observed in the behavioral tests.

We performed quantitative real‐time PCR (qPCR) to measure gene expression levels in hippocampal tissues from each study group. Using the Livak method (see Section 2), we computed the normalized expression levels of the A_2_AR and CB_1_R genes (*Adora2a* and *Cnr1*, respectively) genes to that of the housekeeping gene *Actb* (beta‐actin) for each treatment group relative to their corresponding control group. In parallel, we performed statistical comparisons on the underlying ΔCt values to evaluate whether the observed relative changes reflected statistically significant shifts in expression.

#### 3.2.1 THC Modulates the Adenosinergic and Cannabinoidergic Systems in Anxiety

**FIGURE 4:**
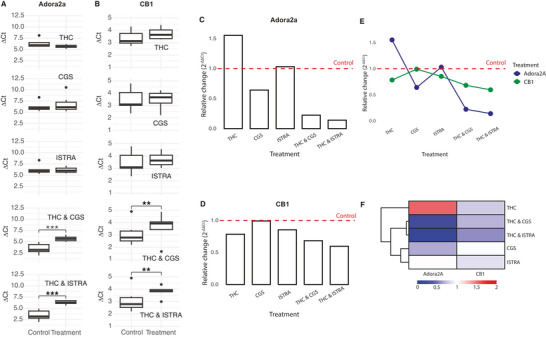
**Gene expression analysis of A_2_AR (*Adora2a*) and CB_1_R (*Cnr1*) in mice hippocampus**. Boxplots show ΔCt distributions for (A) *Adora2a* and (B) *Cnr1* genes for each treatment compared to its respective control (SHAM‐E for THC; SHAM‐D for CGS and ISTRA; SHAM‐E & D for THC & CGS and THC & ISTRA), with individual data points showing outliers. Because ΔCt values are inversely related to transcript abundance, higher ΔCt values correspond to lower gene expression relative to the reference gene. Statistical significance of the test is shown as *p* < 0.05 (*), *p* < 0.01 (**), and *p* < 0.001 (***); nonsignificant comparisons are unlabeled. Normalized relative expression levels of (C) *Adora2a* and (D) *Cnr1* were calculated by the Livak (2^−ΔΔ^ *^Ct^*) method using averaged ΔCt values relative to *Actb* (β‐actin). (E) An interaction plot showing the relative expression differences between the two genes across treatment groups. The red dashed line refers to no change in expression. (F) Visualization of the relative gene expression changes across different treatment groups, hierarchically clustered on the left hand side by the Euclidean distance between groups where value 1 refers to no change and anything below or above refers to a downregulation or upregulation, respectively, relative to the control groups.

The ΔCt comparisons for the single treatment groups (THC, CGS, and ISTRA) versus their respective controls did not reach statistical significance (*p* > 0.05, Figure 4A,B). Nonetheless, the relative changes derived from the Livak method revealed clear directional trends. We observed a sharp increase in the expression of A_2_AR (relative change: 1.55, Figure 4C) and a noticeable decrease in the expression of CB_1_R (relative change: 0.78, Figure 4D) in THC‐treated mice hippocampus, showing THC's effect in both adenosinergic and cannabinoidergic systems (Figure 4E). CGS, a selective A_2_AR agonist, slightly decreased A_2_AR gene expression (relative change: 0.64, Figure 4C), without affecting CB_1_R gene expression (relative change: 0.99, Figure 4D). The inhibitory effects of THC on CB_1_R and CGS on A_2_AR gene expression were as expected through an adaptation mechanism because these substances activate their respective receptors, likely leading to receptor desensitization and/or downregulation through overstimulation. However, THC's modulation of both the adenosinergic and cannabinoidergic systems, particularly supported by anxiety‐like behavior, suggests a potential interplay between these two systems and that THC may play a predominant role in this interaction, either directly through its own receptor activity or indirectly by influencing other components of the adenosinergic–cannabinoidergic signaling network.

#### 3.2.2 ISTRA Maintains A_2_AR Gene Expression and Mildly Downregulates CB_1_R Through Potential Indirect Interactions

ISTRA, a selective A_2_AR antagonist, maintained A_2_AR transcript levels close to control levels (relative change: 1.03, Figure 4C) and slightly reduced CB_1_R expression (relative change: 0.85, Figure 4D). Although these differences were not significant at the ΔCt level (Figure 4A,B), this stabilization indicates that the present blockade of A_2_AR by ISTRA does not trigger compensatory feedback mechanisms to upregulate receptor expression.

#### 3.2.3 Complex Interplay Between A_2_AR and CB_1_R Under Combined Treatments Suggests Potential Heterodimerization

The combination of THC with CGS or ISTRA caused a significant decrease in the expression levels of both A_2_AR (relative change: 0.22 for THC & CGS and 0.14 for THC & ISTRA) and CB_1_R (relative change: 0.68 for THC & CGS and 0.59 for THC & ISTRA), and this decrease was significantly more noticeable in A_2_AR expression than in CB_1_R (Figure 4C,D). In contrast to single treatments, these combined treatments produced statistically significant differences in ΔCt values compared to their respective controls (*p* < 0.01, Figure 4A,B), confirming the observed expression changes represent robust shifts in mRNA abundance. Hierarchical clustering of Livak relative changes based on Euclidean distance branched THC separately from all other treatments. Although individual treatments of CGS and ISTRA clustered together, combined treatments THC & CGS and THC & ISTRA shared the same cluster (Figure 4F). This suggests that, although THC shows a separate expression behavior than other measurements, its combination with CGS or ISTRA is closer to each other than they are to individual treatments of CGS and ISTRA, respectively.

## 4 Discussion

The adenosinergic and cannabinoidergic systems are known to play key roles in regulating anxiety‐like behaviors. However, the molecular interplay between these two systems in modulating anxiety remains underexplored. To address this, we combined behavioral and molecular analyses to investigate the effects of THC, together with the agonist and antagonist of the adenosinergic system, CGS and istradefylline, respectively, on both anxiety‐like behaviors and hippocampal gene expression. Our findings showed that THC and/or CGS induced anxiety‐like behaviors in mice, whereas ISTRA diminished these effects. Notably, THC upregulated adenosine A_2_AR expression and caused a mild reduction in CB_1_R expression in the hippocampus, suggesting that THC's modulation of anxiety‐like behaviors may arise from its ability to influence both systems. These results provide new evidence of an intricate molecular interplay between the adenosinergic and cannabinoidergic systems, connecting hippocampal gene expression changes to behavioral outcomes in mice.

Anxiety is a complex emotional state integrating neurocognitive and sensory processing (Adhikari et al. 2015). In the case of pathological anxiety or anxiety disorders, dysregulation occurs in specific brain regions, such as hyperactivation of the amygdala, hippocampus, thalamus, and cingulum, as well as decreased interconnectivity between these regions (Brooks and Stein 2015; Lai and Wu 2016). As indicated by epidemiological studies, approximately half of the participants in survey studies have reported using cannabis instead of prescription medications to manage anxiety (Turna et al. 2019; Corroon et al. 2017). THC has been shown to induce symptoms such as anxiety, paranoia, depersonalization, and time distortion, which resemble those seen in schizophrenia and other psychotic disorders, even in healthy individuals (D'Souza et al. 2004). Furthermore, it has been shown that the level of anxiety in individuals using THC increases dose‐dependently compared to control groups (D'Souza et al. 2008). In contrast to the anxiogenic effects of THC, studies also indicate that CBD in the cannabis content exhibits anxiolytic effects (Bergamaschi et al. 2011). Cannabis strains with balanced THC and CBD content cause less anxiety than THC‐dominant strains, highlighting the modulatory role of CBD on THC‐induced anxiety (Hutten et al. 2022). However, THC content in the cultivated cannabis plant has increased (Chandra et al. 2019). Therefore, studies on the effects of THC constitute an important area of interest. Additionally, the perspective that the adenosinergic system could be involved in the physiopathology of anxiety‐like behaviors has gained significant importance in recent years (Keyvanloo Shahrestanaki et al. 2024; van Calker et al. 2019; Yamada et al. 2014). Our study contributes to this understanding by focusing on the interplay between the adenosinergic and cannabinoidergic systems and examining THC's role in modulating anxiety‐like behaviors and associated molecular pathways in mice.

The effects of THC in anxiety‐like behaviors are dose‐dependent and vary across animal models. In rats, THC dose has been shown to cause varying effects; for instance, Fokos and Panagis (2010) observed that intraperitoneal THC administration at low doses induced anxiogenic effects, whereas higher doses had anxiolytic outcomes later Rock et al. (2017) reported that 10 mg/kg THC has anxiogenic effects. In mice, the general observation is that low doses of THC produce anxiolytic effects, whereas higher doses lead to anxiogenic outcomes. Kasten et al. (2019) and Valjent et al. (2002) demonstrated that administering THC at 0.3 mg/kg for 5 days induced anxiolytic‐like behavior in mice, whereas a higher dose of 5 mg/kg over the same duration elicited anxiogenic effects. In a study by Kasten et al. (2019), a dose‐dependent anxiogenic effect of THC was shown at 1.0, 5.0, and 10 mg/kg doses, with high doses inducing anxiogenic effects in mice. Similar findings were reported also by Bruijnzeel et al. (2016) and Klein et al. (2011), showing a progressive increase in anxiogenic outcomes with increasing doses in mice, with 10 mg/kg consistently inducing a robust anxiogenic effect. Chronic administration of THC further amplifies these effects; as shown in the study by Murphy et al. (2017), administering THC at 10 mg/kg over an extended period resulted in significant anxiety‐like behaviors in mice. These findings highlight the importance of dose and species‐specific factors in determining THC's behavioral effects. Although lower doses (e.g., 1–5 mg/kg) can also induce anxiety in certain rodent species, higher doses (e.g., 10 mg/kg) have repeatedly been shown to be more reliable for establishing an anxiogenic response and for detecting subsequent molecular adaptations. Given the evidence, we applied a high dose of THC (10 mg/kg) for 5 days in our study on mice to reliably induce anxiety‐like behavior and to detect possible changes in adenosine A_2_AR and cannabinoid CB_1_R gene expression. Anxiety in rodents is commonly assessed using the OF and EPM tests, which evaluate exploratory behavior and anxiety‐related responses. Consistent with prior findings (Kasten et al. 2019; Valjent et al. 2002), we observed that our high‐dose THC‐treated mice exhibited significant anxiety‐like behaviors, measured by reduced center crossings in the OF test and decreased time spent in the open arms alongside increased time in the closed arms of the EPM test. These results confirm the anxiogenic effects of high‐dose THC in our experimental conditions and align with its previously described dose‐dependent effects in mice.

In our study, CGS, a selective A_2_AR agonist, showed a notable anxiogenic effect in behavioral assessment in mice, aligning with its pharmacological action. CGS activates A_2_AR, which are known to enhance glutamatergic signaling and inhibit dopamine D_2_ receptor activity in key brain regions such as the striatum and prefrontal cortex. This mechanism may underlie the anxiety‐like behaviors observed in CGS‐treated mice. Notably, when CGS was combined with THC, the anxiety‐like effects were amplified, suggesting a synergistic interaction between A_2_AR activation and CB_1_R modulation.

Current research on istradefylline has primarily focused on Parkinson's disease (Bara‐Jimenez et al. 2003; Mori et al. 2022; Schwarzschild et al. 2006), highlighting its positive effects on mood disorders such as depression and apathy in these patients. For instance, a study by Nagayama et al. (2025) demonstrated that istradefylline improved symptoms of depression and apathy in Parkinson's patients, independent of its effects on motor symptoms. However, the number of studies on the effects of istradefylline on anxiety remains limited. In our study, we showed that, unlike THC and/or CGS, istradefylline exhibited a calming effect in mice with increased exploratory behaviors in the OF test and decreased anxiety‐like behaviors in the EPM test, in addition to mitigating anxiety‐like behaviors induced by THC.

Our findings are supportive of previous studies suggesting adenosine A_2_AR may contribute to the anxiogenic effects of THC. Adenosine A_2_AR and cannabinoid CB_1_R are found in common regions of the CNS, including the striatum, hippocampus, and cerebellum (Herkenham et al. 1990; Svenningsson et al. 1999). However, the role of these receptors in both the behavioral effects of THC and the role of the adenosinergic system in the effects of THC in the hippocampus is not yet well understood. Following the mostly expected behavioral outcomes of THC and/or CGS or ISTRA, we next investigated the effect of THC treatment in the molecular interplay between the adenosinergic and cannabinoidergic systems in mice hippocampus under THC treatment following behavioral tests. THC and CGS caused a notable decrease in the expression levels of their respective receptor genes, CB_1_R (*Cnr1*) and A_2_AR (*Adora2a*), respectively, and this was as expected due to receptor downregulation through overstimulation. However, THC additionally increased A_2_AR gene expression, exhibiting a broader effect beyond its own receptor gene. This suggests a potential interplay between the adenosinergic and cannabinoidergic systems, and that THC may play a predominant role in this interaction, either directly through its own receptor activity or indirectly by influencing other components of the adenosinergic–cannabinoidergic signaling network. ISTRA maintained A_2_AR gene expression levels close to control levels, indicating that ISTRA does not trigger compensatory feedback mechanisms to upregulate receptor expression as THC and CGS do on their respective receptors. It, however, caused a mild downregulation of CB_1_R gene expression, a surprising phenomenon because CB_1_R is not directly linked to ISTRA's primary pharmacological action. This effect could be due to an indirect cross talk between the A_2_A and CB_1_ receptors as they are part of the same neuromodulatory network in the brain. As CB_1_ receptors can form functional complexes with A_2_A receptors in some brain regions, ISTRA may indirectly alter CB_1_R signaling or expression through shared downstream pathways by modulating A_2_AR activity, even though it does not cause an upregulation of the *Adora2a* expression.

Over the past years, a notable research topic has been the close relationship between the endocannabinoid and adenosinergic systems, and the idea that receptors can form heterodimers (Aso et al. 2019; Carriba et al. 2007; Ferré et al. 2007; Navarro et al. 2008). Indeed, the patterns observed in the combined treatments in our study, particularly the pronounced downregulation of A_2_ARs compared to CB_1_R, are unexpected based solely on the individual effects of these treatments, highlighting the presence of a complex interplay between the adenosinergic and cannabinoidergic systems that cannot be attributed to straightforward pharmacodynamic or physiological interactions. Instead, they may be mediated by heterodimerization and the formation of receptor complexes between A_2_AR and CB_1_R, particularly in brain regions like the hippocampus (Aso et al. 2019). These heterodimers allow the two systems to modulate each other's activity, leading to nonlinear and context‐dependent outcomes. The combination treatments, THC & ISTRA and THC & CGS, likely exacerbate these effects through their opposing actions on the shared signaling pathways of these receptor complexes. THC, as a CB_1_R agonist, modulates downstream pathways like cAMP signaling, whereas CGS and ISTRA, acting on A_2_AR, influence the same pathways in opposing ways (CGS activates A_2_AR, increasing cAMP, whereas ISTRA antagonizes A_2_AR, decreasing cAMP) (R. W. Brown et al. 2020; Cheng et al. 2002; Liu et al. 2024). These opposing forces within the heterodimerized receptor complexes might destabilize the systems’ balance, possibly leading to the downregulation observed in both receptors (Köfalvi et al. 2020). To validate these interpretations, further studies are required. Techniques, such as co‐immunoprecipitation or proximity ligation assays, could confirm the presence and modulation of A_2_AR‐CB_1_R heteromers in the hippocampus under different treatment conditions. Additionally, advanced imaging and molecular biology approaches could help clarify how these receptor complexes contribute to the observed behavioral and molecular outcomes. Understanding these dynamics would provide deeper insights into the role of heteromerization in mediating the interplay between the adenosinergic and cannabinoidergic systems, as well as THC's predominant influence on this interaction.

Our findings on THC treatment in relation to the selective agonist and antagonist of the adenosinergic system showed that a high dose of THC induced an anxiogenic effect confirmed by behavioral tests, and this effect involves an increase in the expression levels of the A_2_AR gene (*Adora2a*) in mice hippocampus.

## Author Contributions

Burçin Ün, Zeki Akarsakarya, and Özlem Yorulmaz Özü performed all experiments, animal procedures, and tissue extraction and wrote the manuscript. Nermin Seda Ilgaz and Mehmet Bertan Yilmaz performed the molecular experiments. Deniz Seçilmiş performed the statistical analysis and visualized the results. Mehmet Ata Seçilmiş designed and supervised the study, revised the manuscript, and provided funding. The final manuscript was reviewed and approved by all authors. The authors declare that all data were generated in‐house, and no paper mills were used.

## Funding

This study was supported by a research grant (TDK‐2021‐14114) from the Scientific Research Projects Unit of Cukurova University.

## Ethics Statement

The experimental protocols were approved by the Ethics Committee of Çukurova University Medical Sciences Experimental Application and Research Center. Procedures in the study were performed in accordance with the NIH Guide for the Care and Use of Laboratory Animals (date: March 18, 2021, and decision number: 11/3).

## Conflicts of Interest

The authors declare no conflicts of interest.

## Data Availability

Data will be made available upon request.
